# Dynamic analyses reveal cytoprotection by RPE melanosomes against non-photic stress

**Published:** 2011-11-09

**Authors:** Janice M. Burke, Patrycja Kaczara, Christine M. B. Skumatz, Mariusz Zareba, Michael W. Raciti, Tadeusz Sarna

**Affiliations:** 1 Department of Ophthalmology, Medical College of Wisconsin, Milwaukee, WI; 2 Department of Biophysics, Faculty of Biochemistry, Biophysics and Biotechnology, Jagiellonian University, Krakow, Poland

## Abstract

**Purpose:**

Isolated melanosomes are known to have antioxidant properties but whether the granules perform an antioxidant function within cells is unclear. The aim of this study was to determine whether retinal pigment epithelium (RPE) melanosomes are competent to protect cultured cells against non-photic oxidative stress induced by treatment with H_2_O_2_.

**Methods:**

Porcine melanosomes, either untreated or irradiated with visible light to simulate age-related melanin photobleaching, were introduced by phagocytosis into ARPE-19 cells. Cells were treated with H_2_O_2_ using two delivery methods: as a pulse, or by continuous generation following addition of glucose oxidase to the medium. Cell survival in melanosome-containing cells was compared to survival in cells containing phagocytosed control latex beads using two real-time cell death assays.

**Results:**

Following H_2_O_2_ delivery by either method, greater resistance to critical concentrations of H_2_O_2_ was seen for cells containing melanosomes than for cells containing beads. Melanosome-mediated protection manifested as a delay in the time of onset of cell death and a slower rate of cell death over time. Photobleaching diminished the stress resistance conferred by the pigment granules. Individual cells in co-cultures were differentially sensitive to oxidative stress depending upon their particle content. Additional features of the time course of the cell death response were revealed by the dynamic analyses conducted over hours post oxidant treatment.

**Conclusions:**

The results show, for the first time, that melanosomes perform a cytoprotective function within cultured cells by acting as an antioxidant. The outcomes imply that melanosomes perform functions within RPE cells aside from those related to light irradiation, and also suggest that susceptibility to ubiquitous pro-oxidizing agents like H_2_O_2_ is partly determined by discrete features of individual RPE cells such as their granule content.

## Introduction

The retinal pigment epithelium (RPE) monolayer is believed to accumulate oxidative damage with aging that leads to diminished support for retinal photoreceptors, thereby contributing to age-related macular degeneration (AMD) [1,2]. To protect against a lifetime of oxidative stress, RPE cells have multiple well-recognized antioxidant protective systems [3], as well as a more controversial potential antioxidant in the form of the pigment melanin. In skin, melanin acts as a photoprotective substance due to its ability to absorb light [4-7] and it likely performs a similar function in the RPE. However, model systems of synthetic melanin or isolated melanosomes have indicated that melanin may have several antioxidant properties in addition to its activity as an optical screen. These properties include the ability to scavenge free radicals [8-10], quench singlet oxygen [11] and the excited states of photosensitizing dye molecules [12-14], inhibit lipid peroxidation [15-17], and sequester multivalent transition metal ions, thereby reducing their availability to participate in reactions that generate strongly oxidizing species [18,19]. Although melanin's function is usually considered to be protection against light irradiation, many of its antioxidant properties could also protect pigmented cells against reactive oxygen species produced in non-photic pathways.

Isolated melanosomes and synthetic melanin clearly display antioxidant properties. Nevertheless, it is unclear whether melanosomes actually perform an antioxidant function in pigmented cells like those of the RPE. Unlike most putative antioxidants, melanin does not exist as a widely distributed soluble factor within cells; instead, it is sequestered in granules whose numbers vary among RPE cells. Melanin can therefore affect only those short-lived reactive species that are generated very near the granule, and if they are to confer significant stress protection, the granule number must be sufficiently high within individual cells to shift the redox environment.

The localization of melanin within melanosomes makes it difficult to detect antioxidant activity by conventional protocols [20]. Stress protection studies on melanosomes have also focused on light-induced stress. This focus is understandable given the location of the RPE, but light stress creates another problem for detecting antioxidant potential; namely, that melanin is able to photo-generate superoxide anion and hydrogen peroxide [8,21-23]. Therefore, upon light irradiation, melanosomes can exhibit a competing function and can act transiently as a pro-oxidant rather than as an antioxidant. Indeed, when the effects of optical screening were carefully controlled in lethal light stress experiments, by comparing cultured cells containing melanosomes to cells containing black particles that could equivalently screen incident light, the cells containing melanosomes were more, not less, susceptible to light damage [24].

Experiments that involve exposure to levels of light sufficient to kill cultured RPE cells within a few hours may preferentially reveal a pro-oxidizing effect of melanosomes, but they do not rule out an antioxidant function of the granules, which could be revealed under conditions of stress from other sources. A ubiquitous source of oxidative stress to cells that reside in well-oxygenated environments is H_2_O_2_, which is generated as a consequence of aerobic metabolism. Although not highly reactive itself, H_2_O_2_ can be converted to the very damaging hydroxyl radical by the Fenton reaction in which iron acts as a co-factor [25]. Iron levels are elevated in eyes with AMD [26], making this metal ion of particular interest to the pathobiology of the RPE. Since melanin can bind iron [27-29] and thereby perhaps affect its availability to support the Fenton reaction, we asked here whether RPE melanosomes are competent to protect cells from H_2_O_2_ exposure.

To conduct the analyses, pigment granules or control particles were delivered to ARPE-19 cells for uptake by phagocytosis and the sensitivity of cells to H_2_O_2_ treatment was then tested using two dynamic assays to evaluate cell death over time. One assay quantifies death in the entire cell population and the other, which involves live cell imaging, quantifies death in cell sub-populations selected for their particle content. Two methods for H_2_O_2_ delivery were also used. One is the conventional method whereby H_2_O_2_ is added to culture medium at moderately high concentrations as a single pulse. In the alternative protocol, glucose oxidase (GOx) is added to the medium to provide a continuous enzymatic generation of H_2_O_2_ from culture medium glucose. We [30] and others [31,32] have previously argued that this alternative represents a sustained delivery method that may be more physiological. The intent was to create assay conditions that might permit detection of what was anticipated to be a small antioxidant effect. Using these assays, melanosomes were shown here, for the first time, to perform an antioxidant function within cells by conferring stress protection against H_2_O_2_-induced toxicity. Also shown was a decline in cellular protection against H_2_O_2_ when granules were experimentally photobleached before uptake by the cells, a protocol used to simulate age-related melanin photo-oxidation [29]. The dynamic assays also revealed other features of the cellular response to H_2_O_2_ treatment including a surprisingly long delay in the onset of cell death after pulse addition of low oxidant doses, differing patterns of cell death that depended upon when critical killing doses were achieved, and outcomes that suggested that susceptible cells in the monolayer may adversely affect the survival of adjacent cells.

## Methods

### Melanosome preparation, cell cultures, and particle phagocytosis

Melanosomes were purified from the RPE of porcine eyes as previously described [24,33] including the step in which isolated granules were detergent treated to remove membranes and contaminating material associated with the granule surface [34]. For some experiments, melanosomes were photobleached by irradiating granules with intense visible light, as previously described [29], to simulate changes that may occur in pigment granules during RPE cell aging.

The human RPE cell line ARPE-19 (American Type Culture Collection, Rockville, MD) was used for oxidative stress analysis. The cultures were propagated in Minimal Essential Medium (MEM) containing 10% fetal bovine serum and loaded with particles by phagocytic uptake over 24 h, using purified porcine melanosomes prepared as described above or 1µm black latex beads (Invitrogen, Carlsbad, CA) as a phagocytosis control. The methods for particle uptake, which were previously reported [20,24], produce comparable particle numbers per cell for all particle types. After phagocytosis, the particle-loaded cells were re-plated in assay wells (96 well plates or 8-chamber slide wells) at a density that produced confluency after cell attachment and spreading (~1×10^5^ cells/cm^2^). Experiments involving H_2_O_2_ treatment were initiated one to five days later.

When imaging methods were used for analysis of H_2_O_2_-induced cytotoxicity (see following sections), a co-culture strategy was used to compare the effects of different particle types within adjacent cells in the same monolayer. To produce the co-cultures, paired populations of cells phagocytosed the particle types slated for comparison, and the two populations of cells were then re-plated in a 1:1 ratio in the multi-chamber slide wells used for imaging. In co-cultures of cells containing either melanosomes or control latex beads, the cells containing beads were distinguished from those containing melanosomes by the endogenous autofluorescence of the beads (635/685 excitation/emission). In co-cultures of cells containing either untreated or photobleached melanosomes, the granule content of each cell was discriminated by spiking the granules before phagocytosis with small numbers of two different types of 1µm fluorescent beads (ThermoScientific, Palo Alto, CA), which were detected using their specific excitation/emission wavelengths (542/612 or 468/508).

### Stress induction with hydrogen peroxide

To test for H_2_O_2_-induced cytotoxicity in particle-loaded ARPE-19 cells, two methods for oxidant delivery were used according to our published techniques [20,30]: pulse delivery or continuous generation with glucose oxidase. Briefly, for pulse delivery, a range of concentrations of freshly prepared H_2_O_2_ (0–2 mM) was added to the cultures. For continuous enzymatic generation of H_2_O_2_, the cultures were re-fed with fresh glucose-containing solutions and GOx was added to produce final enzyme concentrations of 0–40 mU/ml.

### Dynamic assays of H_2_O_2_-induced cytotoxicity

Two dynamic cytotoxicity assays were used, both of which employed addition of the membrane impermeant fluorescence dye propidium iodide (PI; Sigma, St. Louis, MO) to the cultures. Nuclear fluorescence in cells with damaged membranes resulting from H_2_O_2_ treatment was then quantified over time. For both assays, PI was added to the cultures at a final concentration of 100 μM approximately 30 min before the addition of H_2_O_2_ (pulse delivery) or of GOx (continuous H_2_O_2_ generation). PI fluorescence was measured at intervals after oxidant addition using a plate reader for cell populations, or by live cell imaging for individual cells.

For the assay of cell populations, cells were treated with H_2_O_2_ in replicate wells of 96-well plates in the presence of PI dissolved in Hank’s Balanced Salt Solution with calcium and magnesium ions (HBSS; Invitrogen, Carlsbad, CA). When GOx was used to generate H_2_O_2_, the HBSS also contained 1 mg/ml glucose. Plates were immediately placed in the chamber of a Synergy H4 plate reader (Biotek Instruments, Winooski, VA) at 37 °C and PI fluorescence (555/617 nm excitation/emission) was recorded in arbitrary units at 10 min intervals for up to 20 h. The means (±SD) of the fluorescence readings from replicate wells were calculated using Microsoft Excel (Microsoft Corporation, Seattle, WA). Fluorescence intensity data were plotted versus time and curve fitting and regression analyses were performed using Origin software (OriginLab, Northampton, MA). Comparisons between cell populations containing different particle types were made within experiments using culture wells in the same plate. Each comparison was made from a minimum of three independent experiments. Three components of the curves for PI fluorescence intensity (time of onset, slope and peak fluorescence) were separately analyzed to identify differences between H_2_O_2_ concentrations, as explained further in the Results.

For the imaging assay, co-cultures were treated in replicate wells of chamber slides in the presence of PI dissolved in serum-containing culture medium. Slides were mounted on the stage of a microscope equipped with an environmental chamber to control temperature, humidity, and CO_2_ levels, as previously described [24]. Digital bright-field and fluorescence images (to detect PI and latex beads) were captured sequentially at 5 min intervals for up to 18 h in four fields per culture well. Premier MetaMorph software (Molecular Devices, Sunnyvale, CA) was used for image capture and data analysis. For analysis, bright-field images were used to pre-select cells with comparable particle numbers and overlays of bright-field and bead fluorescence images were used to identify the particle types in each selected cell, as explained further in the Results. Overlays were produced of the bright field and PI fluorescence images captured over time and were assembled to generate time-lapse movies. Comparisons between cells containing different particle types were made for cells within the same co-culture by conducting frame-by-frame analysis to identify the time when the PI staining in the nucleus of each pre-selected cell achieved threshold fluorescence. Data were plotted as the percent of surviving cells (no nuclear PI) in the pre-selected cell population over time. Significant differences were determined using GraphPad Prism 5 survival analysis (GraphPad Software, Inc., La Jolla, CA). Each comparison was made on a minimum of three independent experiments.

## Results

### Dynamic H_2_O_2_ cytotoxicity assay of ARPE-19 populations

**Figure 1:**
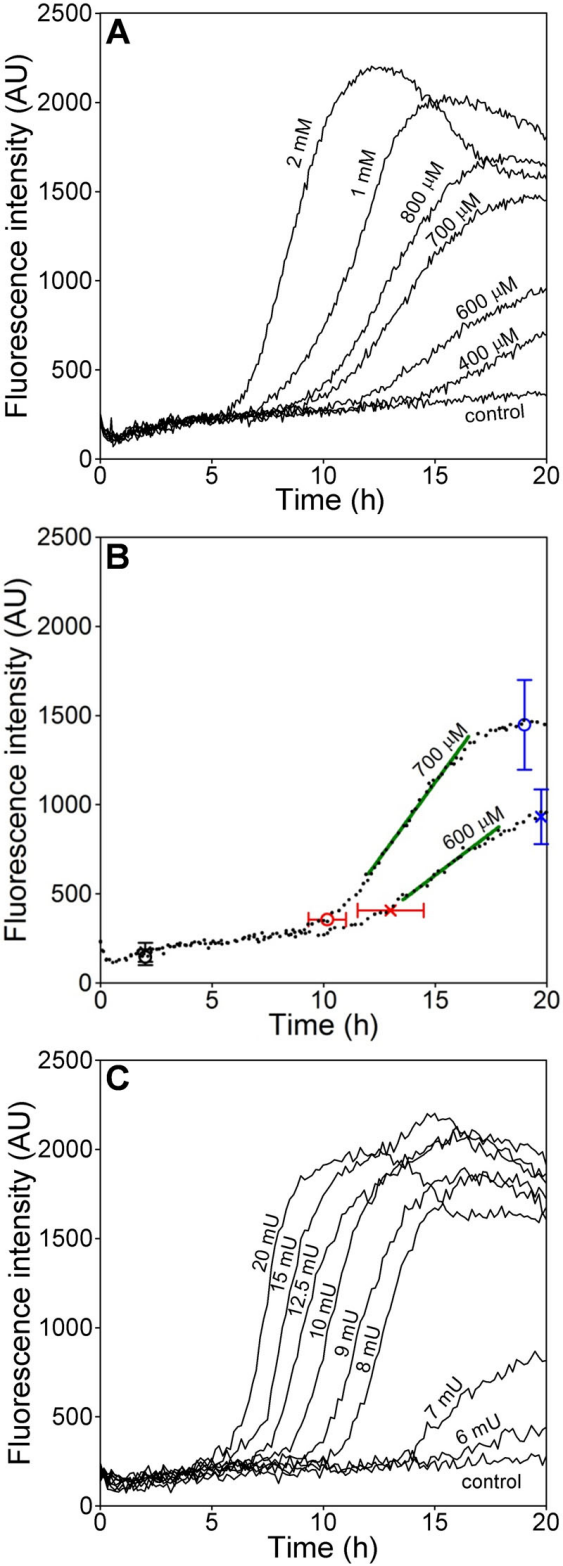
Dose-dependent H_2_O_2_-induced toxicity in ARPE-19 cell populations shown by dynamic changes in propidium iodide (PI) fluorescence. PI fluorescence (in arbitrary units) was measured over 20 h in replicate wells of ARPE-19 cultures exposed to varying amounts of H_2_O_2_. **A**: H_2_O_2_ delivered as pulse at the concentrations indicated. **B**: Isolates three components of the curves for PI fluorescence intensity that were used for comparison between treatment groups, using 600 (X's) and 700 μM (circles) H_2_O_2_ for illustration. The times of onset of the fluorescence rise (red) relative to baseline at 2 h (black), the mean slopes (green), and the peak fluorescence values (blue) were determined as described in Table 1. **C**: H_2_O_2_ generated enzymatically by the addition of glucose oxidase (GOx) at the mU/ml concentrations indicated. Data in **A** and **C** are the means of 3–6 replicate culture wells per group within representative experiments.

An assay method was developed in which the time-dependent generation of PI fluorescence was quantified in ARPE-19 cells exposed to H_2_O_2_ (Figure 1). When the oxidant was delivered as a pulse, a sensitive dose dependency across a range of concentrations was found, as illustrated for doses between 400 μM and 2 mM H_2_O_2_ (Figure 1A). A time lag of several hours was observed after oxidant addition before the fluorescence began to increase, and the time of onset of the fluorescence rise was progressively delayed as the oxidant dose decreased. After this lag phase, the slopes of the lines describing increasing fluorescence differed depending on oxidant concentration, as did the peak intensity that was achieved during the experiment. These three components of the curves for PI fluorescence intensity (time of onset, slope, and peak fluorescence) were separately analyzed to identify significant differences between H_2_O_2_ concentrations. As an example, comparing outcomes for two similar concentrations (600 and 700 μM) indicates that the time of onset, mean slope, and peak fluorescence all differed significantly (Figure 1B and Table 1). For cultures exposed to higher concentrations of oxidant and exhibiting earlier cell death, fluorescence intensity often declined after peaking (e.g., see 2 mM H_2_O_2_ in Figure 1A). The shape of the curves after peaking, which was complex and likely due to several factors including fading of PI fluorescence in cells with early onset death, was not used for comparison among treatment groups.

**Table 1:** Comparison of the features of the dynamic curves for PI fluorescence in ARPE-19 cultures illustrated in Figure 1 after pulse delivery of H_2_O_2_ or after enzymatic generation of H_2_O_2_ by glucose oxidase (GOx) addition.

**Pulse delivery**				**Enzymatic generation**			
**H_2_O_2_** **(μM)**	**onset time (h±SD)**	**mean slope**	**peak intensity (AU±SD)**	**GOx (mU/ml)**	**onset time (h±SD)**	**mean slope**	**peak intensity (AU±SD)**
0				0			
400	15.6±1.3*	69*	681±99*	6	19.8±1.0*	56*	512±71*
600	13.2±1.5*	86*	931±153*	7	14.7±1.7*	97*	881±170*
700	10.2±0.8*	146*	1446±251*	8	11.7±0.4*	381*	1838±147*
800	10.0±0.8	213*	1653±215	9	10.5±0.6*	367	1911±149
1000	8.6±0.8*	329*	1987±179*	10	9.2±1.0*	442*	2165±240*
2000	6.5±0.4*	459*	2199±165*	12.5	8±0.4*	425	2155±74
				15	7.2±0.5*	628*	2248±112
				20	6±0.3*	573	1966±139*

Onset time is the time post-addition of H_2_O_2_ or GOx that PI fluorescence intensity increased by 5 standard deviations above baseline (defined as intensity at 2 h). The mean slope (M in the equation Mx+b) is for the line describing the increasing intensity during the linear phase of the rapid rise in fluorescence intensity as illustrated in Figure 1B (in green). The peak intensity (in arbitrary units [AU]) is the highest fluorescence intensity achieved during 20 h. Asterisks indicate where the outcome differs significantly (paired *t* test, p<0.02) from the outcome obtained for the same parameter in the next lower concentration of H_2_O_2_ or GOx.

A dose dependency was also observed when H_2_O_2_ was generated enzymatically by the addition of GOx to the cultures (Figure 1C), but the kinetics of the increase in PI fluorescence intensity differed from kinetics seen in experiments employing pulse H_2_O_2_ delivery. After GOx addition, the time lag until the onset of apparent cell death increased with decreasing enzyme concentration (Figure 1C), likely reflecting different times required for H_2_O_2_ to achieve critical toxic levels, as shown previously [30]; this made the time of onset a sensitive discriminator of dose-dependent cytotoxicity (Table 1). Thereafter, at higher concentrations of GOx (in the range of 8–20 mU/ml in the experiment shown [Figure 1C]), cell death was rapid, with parallel slopes of the lines for increasing fluorescence intensity. At lower concentrations of enzyme (e.g., 6 and 7 mU/ml in Figure 1C), the rate of cell death was slower, as indicated by the shallower slope of the line for increasing fluorescence. Differing slopes occurred in a narrow range of concentrations in each experiment; added enzyme usually either produced rapid cell death when a critical H_2_O_2_ concentration was reached or produced virtually no cell death. Higher concentrations of enzyme that produced early onset cell death often showed lower peak fluorescence than did lower GOx doses (e.g., compare 20 mU/ml to 10–15 mU/ml, Figure 1C). This result is predictable based on results shown previously that illustrated changes in the culture medium H_2_O_2_ concentration over time [30]. With high GOx, H_2_O_2_ concentrations initially rise rapidly until levels are achieved in the culture medium that are sufficient to kill some cells and to inhibit GOx, which together produce rapid declines in H_2_O_2_ [30]. Peak fluorescence therefore did not discriminate GOx dose and was not used for comparison of treatment groups in subsequent experiments.

Although low doses of GOx or H_2_O_2_ were consistently sub-lethal and high doses were consistently lethal, the doses that generated graded cell death responses varied substantially among experiments; e.g., from approximately 200 μM to 1 mM when H_2_O_2_ was delivered as a pulse (not shown). When testing effects of melanosomes, as described below, a range of concentrations of oxidant or enzyme was used in all experiments. The concentration(s) in each experiment that produced a graded response, permitting detection of either increases or decreases in the dynamic changes in fluorescence intensity, were used for comparing cultures containing different particle types.

### Dynamic H_2_O_2_ cytotoxicity assay of ARPE-19 populations and the effect of melanosomes

**Figure 2:**
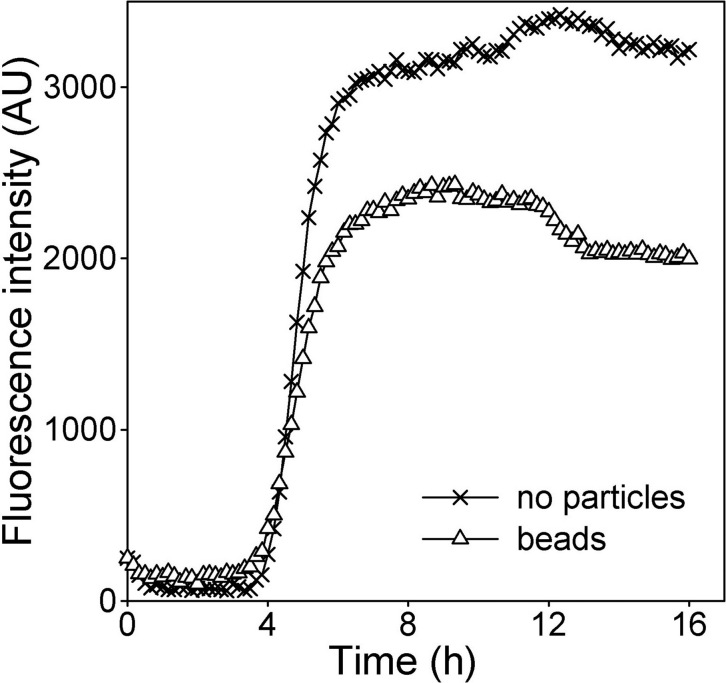
Effect of phagocytosed particles on the sensitivity of ARPE-19 cell populations to H_2_O_2_-induced toxicity. Dynamic changes in propidium iodide (PI) fluorescence (in arbitrary units [AU]) are shown in ARPE-19 cells containing no particles (X's) or pre-loaded by phagocytosis with latex beads (triangles) during treatment with 500 μM H_2_O_2_. For no particle versus bead containing cells, respectively, time of onset of cell death (3.8±0.5 versus 4.2±0.2 h) did not differ, but mean slopes (1630 versus 1013) and peak fluorescence intensities (2430± 394 versus 3422±420) differed significantly (unpaired *t* tests with equal variances, p<0.02).

Prior to testing the effects of melanosomes introduced into ARPE-19 cells by phagocytosis, preliminary experiments were performed to determine whether cells containing phagocytosed control particles (latex beads) differed from particle free cells in their sensitivity to H_2_O_2_–induced stress. The times of cell death onset were similar, but a small, consistent difference was observed in the slopes and peaks of fluorescence intensity in the dynamic PI fluorescence assay, with bead-loaded cells showing greater resistance to oxidant toxicity (Figure 2). To confirm that this outcome was not due to simple blocking of fluorescence by the black beads, experiments were performed using the imaging assay discussed below, where stained nuclei were counted, and similar results were obtained (not shown). Phagocytosis or the presence within cells of phagocytosed particles therefore appeared to have a small stress-protective effect that must be controlled for in experiments designed to determine whether melanosomes confer antioxidant protection. Thus, in experiments where the function of phagocytosed RPE melanosomes was tested, cells containing phagocytosed control particles, rather than particle-free cells, were used as the control population.

**Figure 3:**
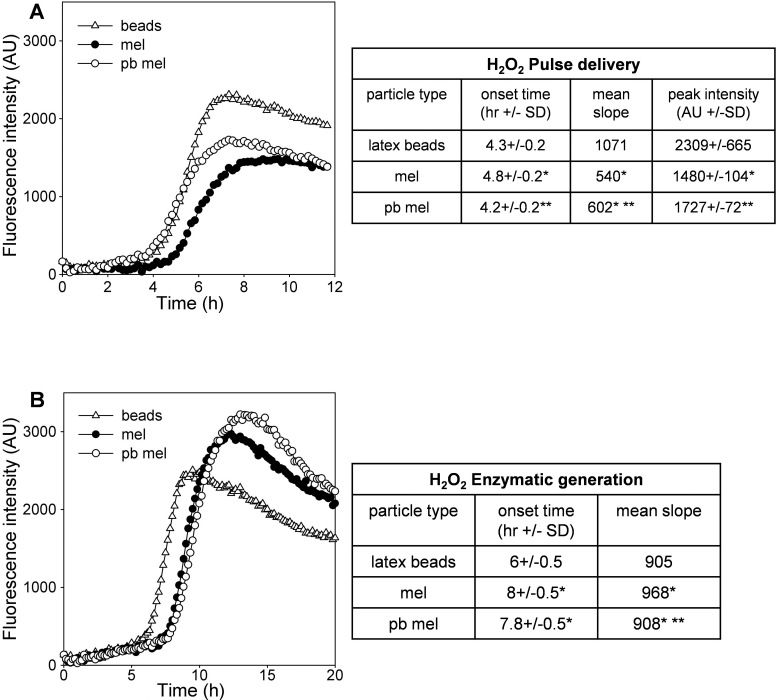
Effect of phagocytosed melanosomes on the sensitivity of ARPE-19 cell populations to H_2_O_2_-induced toxicity. Dynamic changes in propidium iodide (PI) fluorescence (in arbitrary units [AU]) in ARPE-19 cultures treated with **A**: 200 μM H_2_O_2_ or **B**: 16 mU/ml glucose oxidase (GOx) to generate H_2_O_2_ enzymatically. Cells were pre-loaded by phagocytosis with latex beads (triangles), untreated melanosomes (mel, black circles) or photobleached melanosomes (pb mel, open circles). Data are the means of 3 replicate culture wells per treatment group within representative experiments of each type. Accompanying tables show the corresponding quantitative data. Onset times, mean slopes and peak intensities are as described in Figure 1B and Table 1. Single asterisks indicate where the outcome for that parameter differs significantly from the latex bead control; double asterisks indicate where pb mel differs significantly from mel (unpaired *t* tests with equal variances, p<0.02).

ARPE-19 cells pre-loaded with melanosomes by phagocytosis showed a reproducible cytoprotection against H_2_O_2_–induced stress when compared to control cell populations containing phagocytosed latex beads (Figure 3). When H_2_O_2_ was delivered as a pulse (Figure 3A and accompanying table), melanosome-containing cells showed a significantly longer time lag until the onset of cell death, a slower rate of death, and a lower maximal cell death. When H_2_O_2_ was generated enzymatically (Figure 3B and accompanying table), melanosome-mediated cytoprotection manifested as a delayed onset of cell death. Comparison of photobleached melanosomes with untreated granules showed no consistent difference in assays employing GOx (Figure 3B). However, when H_2_O_2_ was delivered as a pulse, a diminished cytoprotective effect of photobleached granules was observed that manifested primarily as a more rapid onset of cell death (Figure 3A and accompanying table).

### Dynamic H_2_O_2_ imaging-based cytotoxicity assay of individual ARPE-19 cells and the effect of melanosomes

**Figure 4:**
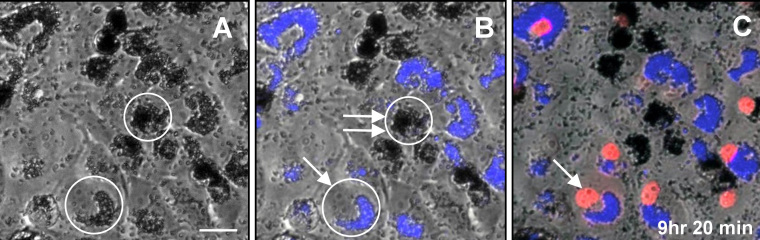
Illustration of an imaging assay using co-cultures of ARPE-19 cells pre-loaded by phagocytosis with control latex beads or melanosomes. The same microscope field is shown by **A:** bright-field microscopy, and **B**, **C**: in merged bright-field and fluorescence images. The fluorescence image overlay in **B** shows latex bead autofluorescence (artificially colorized blue) and the double overlay in **C** also shows PI fluorescence (red), illustrating nuclear staining at 9 h and 20 min after addition of 750 μM H_2_O_2_. Circled cells in **A** show examples of cells pre-selected for analysis by their particle content. In **B,** the same cells are circled to illustrate discrimination of particle type in the selected cells; one contains beads (arrow) and the other melanosomes (double arrow). The arrow in **C** indicates a PI-positive nucleus in the bead-containing cell after H_2_O_2_ treatment. Scale bar: 20 μm.

Since the protective effects of melanosomes were small when analyzed in cell populations as shown above, and since cells vary in number of phagocytosed particles of all types [20], we sought another method to verify the outcome. An imaging-based assay was employed that permits pre-selection of individual cells based upon their particle content so that similarly loaded cells could be compared. The assay is illustrated in Figure 4 for an experiment comparing cells in co-cultures that were pre-loaded with either melanosomes or control latex beads. Co-cultures at confluent density were imaged by bright-field microscopy (Figure 4A) to determine the particle numbers per cell and to allow pre-selection of cells with at least 50 particles for analysis, which was accomplished by particle counts. During H_2_O_2_ treatment, bright field and fluorescence images were captured over time in the presence of PI, as described in the Methods. Fluorescence images were used to detect latex bead fluorescence, thereby discriminating the particle type in each pre-selected cell (Figure 4B), and to detect nuclear PI fluorescence (Figure 4C).

**Figure 5:**
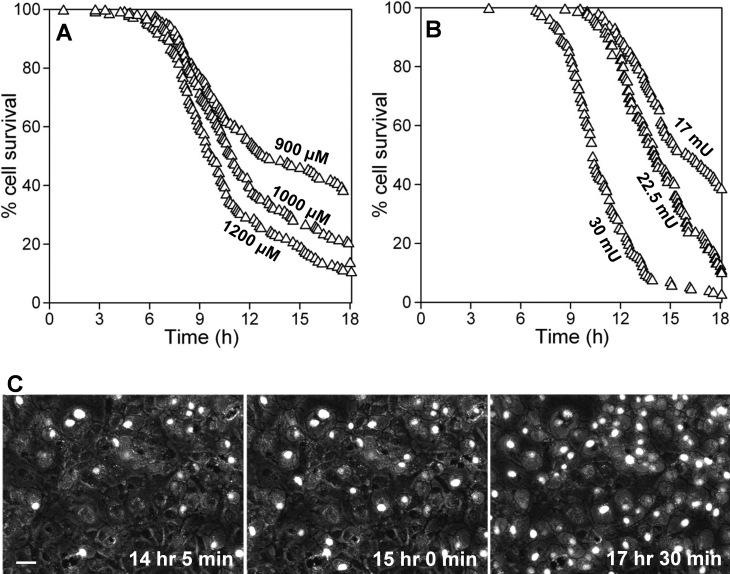
Dose-dependent H_2_O_2_-induced toxicity in individual ARPE-19 cells shown by time-dependent image analysis of nuclear propidium iodide (PI) fluorescence. Live cell imaging of dynamic changes in nuclear PI fluorescence in ARPE-19 cells containing control particles (latex beads) after treatment with three doses of H_2_O_2_ delivered either as **A**: a pulse or **B**: generated enzymatically after the addition of glucose oxidase (GOx) The concentrations of H_2_O_2_ (in μM) or GOx (in mU/ml) are indicated. Numbers of cells selected for analysis were as follows. **A**: 900 μM, n=166; 1000 μM, n=194; 1200 μM, n=163. **B**: 17 mU/ml, n=123; 22.5 mU/ml n=123; 30 mU/ml, n=165. Data are from representative experiments and are the percent of cells pre-selected for bead content that survived (no nuclear PI) with time. All curves within each treatment protocol differ significantly from one another (GraphPad Prism 5 survival analysis, p<0.02). **C**: Bright-field-fluorescence overlays of cultures treated with 22.5 mU/ml GOx to illustrate the time-dependent increase in numbers of cells exhibiting nuclear PI fluorescence (shown white). Images were captured at 5 min intervals; the acquisition times of the selected images are indicated. Scale bar: 20 μm.

Preliminary studies were conducted using cells containing beads to determine the pattern and dose-dependency of cell death induced by H_2_O_2_ treatment in the imaging assay using both pulse delivery and enzymatic generation protocols (Figure 5). As shown, concentrations of H_2_O_2_ could be identified that produced a graded cell death response, detected using the time-dependent acquisition of nuclear PI fluorescence in images. Similar to the cell population-based assay used above, a time lag of several hours was observed before the onset of nuclear PI staining was detected, and the lag was longer with GOx than with pulse H_2_O_2_ delivery, especially at low GOx concentrations. After the onset of cell death , increasing concentrations of H_2_O_2_ or GOx produced steeper slopes of the curves that described the rate of cell death (percent cell survival with time).

**Figure 6:**
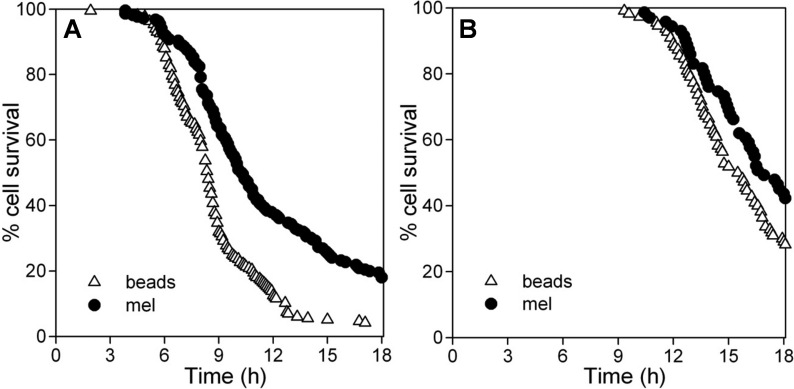
Effect of melanosomes on the sensitivity of individual ARPE-19 cells to H_2_O_2_-induced toxicity compared to cells with a comparable content of control particles (latex beads) in co-cultures. Live cell imaging of dynamic changes in nuclear PI fluorescence in ARPE-19 cells pre-loaded with latex beads or melanosomes (mel) and treated with H_2_O_2_ delivered as **A**: a pulse at 1000 μM or **B**: generated enzymatically by the addition of GOx at 30 mU/ml. Numbers of cells selected for analysis in the same co-culture wells containing the two particle types were as follows. **A**: beads, n=216 (triangles); mel, n=216 (black circles). **B**: beads, n=110 (triangles); mel, n=71 (black circles). Data are from representative experiments and are the percent of the pre-selected cells in each particle group surviving (no nuclear PI) with time. The shift to the right of the curves for mel illustrates the magnitude of the protective effect of mel relative to beads. The curves for the two particle types within each treatment protocol differ significantly (GraphPad Prism 5 survival analysis, p<0.02).

A protective effect of melanosomes was observed for H_2_O_2_ treatment using the imaging method that compared cells in co-cultures containing comparable numbers of either control latex beads or melanosomes (Figure 6). At concentrations of H_2_O_2_ that produced a moderate rate of cell death, cells containing melanosomes exhibited a slower rate when compared to immediately adjacent cells that containing beads. The time of onset of cell death after oxidant addition was similar regardless of the ingested particle type.

**Figure 7:**
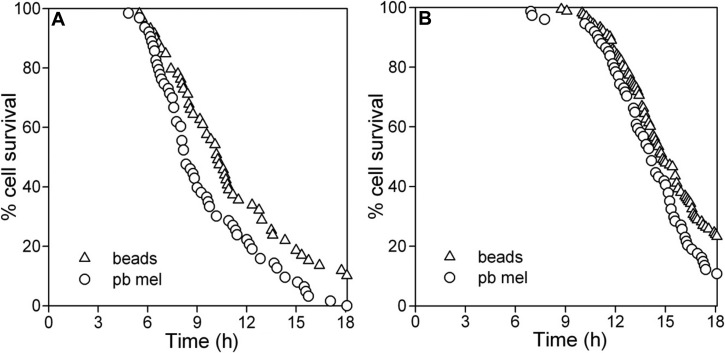
Effect of photobleached melanosomes on the sensitivity of individual ARPE-19 cells to H_2_O_2_-induced toxicity compared to cells with a comparable content of control particles (latex beads) in co-cultures. Live cell imaging of dynamic changes in nuclear PI fluorescence over 18 h in ARPE-19 cells pre-loaded with latex beads or photobleached melanosomes (pb mel) and treated with H_2_O_2_ delivered as **A**: a pulse at 1500 μM or **B**: generated enzymatically by the addition of GOx at 40 mU/ml. Numbers of cells containing the different particle types selected for analysis were as follows. **A**: beads, n=59 (triangles); pb mel, n=63 (circles). **B**: beads, n=163 (triangles); pb mel, n=74 (circles). Data are from representative experiments and are the percent of the pre-selected cells in each particle group surviving (no nuclear PI) with time. The shift to the left of the curves illustrates the magnitude of the cytotoxic effect of pb mel relative to beads. The curves for the two particle types within each treatment protocol differ significantly (GraphPad Prism 5 survival analysis, p<0.02).

**Figure 8:**
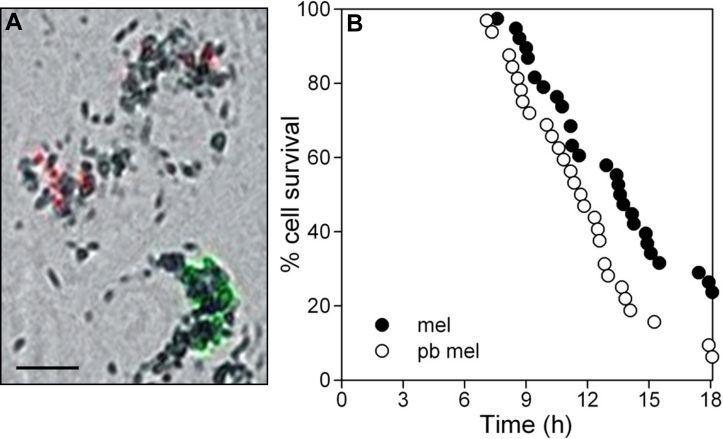
Direct comparison of the effects of photobleached and untreated melanosomes on the sensitivity of individual ARPE-19 cells to H_2_O_2_-induced toxicity in co-cultures. **A**: Merged bright-field and fluorescence images illustrating cells in co-cultures containing either untreated granules (identified by co-phagocytosis of marker fluorescent beads detected by 542 excitation/612 emission [shown red, two upper cells]), or containing photobleached granules (identified by marker beads with 468 excitation/508 emission [shown green, lower cell]). Scale bar: 10 μm. **B**: Live cell imaging of dynamic changes in nuclear PI fluorescence for co-cultured cells pre-loaded with untreated (mel) or photobleached melanosomes (pb mel) and treated with 800 μM H_2_O_2_. Numbers of cells containing the different particle types pre-selected for analysis were as follows: mel, n=38 (black circles); pb mel, n=32 (open circles). Data are the percent of the pre-selected cells in each particle group surviving (no nuclear PI) with time. The shift to the left of the curve for pb mel illustrates the magnitude of the decreased survival for cells containing pb mel relative to mel. The curves differ significantly (GraphPad Prism 5 survival analysis, p<0.02).

Using a similar imaging strategy, ARPE-19 cells containing photobleached melanosomes showed no protection against H_2_O_2_–induced toxicity when compared to adjacent cells containing comparable numbers of beads. In fact, these cells often demonstrated increased rates of cell death (Figure 7). In contrast to the cell population-based assay described above, the ability to focus on the subset of cells with abundant granules by imaging methods permitted detection of diminished cytoprotection by photobleached granules after both pulse H_2_O_2_ delivery (Figure 7A) and GOx treatment (Figure 7B). Loss of protection due to photobleaching was confirmed when cells containing photobleached melanosomes were directly compared with cells containing untreated granules in co-cultures (Figure 8). In this protocol, granule type in adjacent cells was distinguished by the detection of different fluorescent beads that were delivered with the granules during phagocytosis to serve as markers (Figure 8A). Cells containing photobleached melanosomes exhibited a similar time of cell death onset to cells containing untreated granules, but their rate of death was more rapid (Figure 8B).

### Dynamic H_2_O_2_ imaging-based cytotoxicity assay reveals a non-random distribution of dying cells

**Figure 9:**
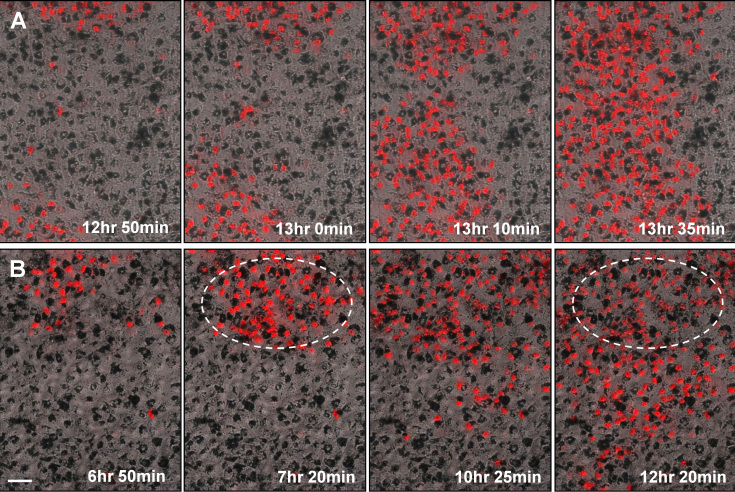
Non-random distribution pattern in the time-dependent acquisition of nuclear propidium iodide (PI) fluorescence in ARPE-19 cells exposed to toxic doses of H_2_O_2_. Live cell imaging of dynamic changes in nuclear PI fluorescence in ARPE-19 cells following pulse delivery of H_2_O_2_. Images are bright-field-fluorescence overlays from two separate experiments illustrating nuclear PI staining (red) in cells at selected time intervals after exposure to row **A**: 750 μM or row **B**: 1000 μM H_2_O_2_. Images were captured at 5 min intervals after oxidant addition; the acquisition times of the selected images are indicated. The circled area identifies a region where earlier onset nuclear PI fluorescence has begun to fade 5 h later. Scale bar: 40 μm.

As indicated above, the kinetics of cell death demonstrated by analysis of PI fluorescence in ARPE-19 cells exposed to H_2_O_2_ showed a several-hour time lag before the onset of death and a long and complex time course for recruitment of cells into the non-surviving population. Another noteworthy observation made by real-time imaging indicated a nonrandom distribution of dying cells in the cultures. Treatment of cells with H_2_O_2_ triggered a pattern of cell death in some microscope fields that spread from an apparent origination site to adjacent cells in the monolayer (Figure 9). Two illustrations are provided. In one, two foci of PI-positive cells spread and converge (Figure 9A). In the other, a focus of PI positive cells spreads from top to bottom across the microscope field (Figure 9B). Also illustrated is the fading of nuclear PI over time in the portion of the field where cell death began hours earlier (Figure 9B). (PI fading helps to explain the declining fluorescence intensity over time after the peak in the population-based assays shown in Figure 1, Figure 2, and Figure 3.)

## Discussion

Despite ample evidence to indicate that melanin has antioxidant properties, prior to the present investigation no convincing data existed to show that pigment granules might protect RPE cells against oxidative stress by acting as an antioxidant. Protection of RPE cells against stress from light irradiation has been observed in vitro by comparing amelanotic to melanotic cells [35,36], but this outcome does not distinguish the pigment’s ability to act as an optical screen from its putative antioxidant properties. When controls for optical absorbance were included, by comparing ARPE-19 cells containing optically dense particles that lack antioxidant function, melanosomes showed no protection against light-induced stress using conventional measures of cytotoxicity [20]. Re-evaluation of lethal photic stress by more sensitive dynamic measures actually revealed increased rather than decreased light toxicity in cells containing melanosomes, which was attributed to the photo-reactive, pro-oxidizing properties of melanin [24].

Regardless of these outcomes, it seemed premature to conclude that melanosomes cannot act as an antioxidant given the limitations in detecting the antioxidant function of a pigment that is confined within granules with a limited sub-cellular distribution and which may protect only the subset of cells that has an adequate granule content. In addition, the use of light as a stressor in the past may have introduced a bias toward detecting the competing pro-oxidizing properties of melanin induced by irradiation. The primary intent of the present investigation therefore was to test whether melanosomes function as a cytoprotective antioxidant in cells exposed to a non-photic form of stress; namely, treatment with H_2_O_2_.

This strategy was attempted previously using conventional single end point measures of toxicity, but no protection against chemical oxidants was detected [20]. In the present study, however, the use of real-time analyses of propidium iodide fluorescence to reveal the kinetics of cell death showed that ARPE-19 cells loaded with melanosomes were more resistant to H_2_O_2_ treatment than were cells loaded with control beads. In the population-based PI fluorescence assay, the presence of melanosomes resulted in delays in cell death similar to those resulting from treatment with a lower H_2_O_2_ dose, which shifted the factors such as time of onset, slope, and peak of the cell death curves. Since cells that survive initial hours of oxidant exposure can continue to survive (not shown), the effect of melanosomes is not only to delay cell death, but to avert it in a subset of cells.

Photobleaching of melanosomes as a way of experimentally aging the pigment granules was observed to reduce their protective capacity. The imaging-based PI fluorescence assay was more effective at revealing this loss of protection and permitted detection of the effect when H_2_O_2_ was delivered as a pulse as well as when the oxidant was generated enzymatically. The increased sensitivity of detection afforded by image analysis likely resulted from the ability for more direct comparison of cells with abundant and comparable particle contents. The mechanism of antioxidant protection by melanosomes is not yet determined, but granule number per cell is expected to play a role in determining whether pigment content shifts the redox environment sufficiently to affect cell survival.

One potential antioxidant mechanism that is currently under investigation is iron binding by melanin [18,19], which has the potential to modulate hydroxyl radical generation via regulation of the Fenton reaction. Iron binding is an attractive mechanism because the magnitude of iron binding within each cell would depend upon its granule number, and the reduction in iron-binding capacity by photobleaching [29] could explain the diminished protective property of photobleached granules. Whether iron binding could fully explain the protective effect of melanosomes is unclear, however. Melanosome-mediated protection occurs even when the onset of cell death is delayed for several hours after exposure to low concentrations of H_2_O_2_, long after H_2_O_2_ in culture medium is depleted (this occurs within 2 h) [30]. This protracted effect raises the possibility that melanosomes have additional unidentified biological functions beyond the transient regulation of the generation of short-lived reactive species. One unexplored possibility is that the presence of melanosomes alters overall iron homeostasis. This could have multiple secondary effects, perhaps including changes in expression levels of relevant cytoprotective proteins such as antioxidant enzymes in the heme oxygenase system [37]. This possibility is also being pursued.

Considerable emphasis was placed in this investigation on describing the real-time measures of PI fluorescence in living cells subjected to oxidative stress. Related methods have been used previously, including by us [24], and the method itself was recently evaluated [38]. In the present study, we used rapid data acquisition intervals (minutes) over extended time frames (hours) to examine the kinetics of cell death after H_2_O_2_ treatment and found that dynamic measures were highly sensitive for discriminating dosage effects of this commonly-used oxidant. Doses at the borderline of lethality were the most useful for identifying small antioxidant effects such as those mediated by granules, but due to the sensitivity of the assay, the critical doses were difficult to predict across experiments. Indeed, the inter-experiment variability was perhaps surprisingly high, which merits additional comment. Conventional measures of cell death, such as those used by us in other investigations [20], employ fixed end-point analyses that do not reveal differences in the timing of cell death that occurs at different oxidant doses. In the present case, we titrated the dose of oxidant to find a concentration in each experiment that stratified cells into susceptible and resistant populations over a short window of time in the dynamic assays. In our experience, small manipulations of cultures could affect this critical dose, including seemingly inconsequential handling methods like culture feeding schedules or time of day when assays are performed. Although this sensitivity can make these experiments challenging, it can also reveal small antioxidant effects provided that rigorous controls are employed within experiments and consistent outcomes are found across experiments (in our case, showing that melanosomes protect). This overall strategy may be useful for detecting other weak antioxidants as well.

The kinetic measures of cell death in experiments using high concentrations of GOx showed complicated outcomes. This might be expected given that medium levels of H_2_O_2_ fluctuate with high GOx, initially rising, then holding steady when generation balances depletion, and finally falling as products released from dying cells accelerate H_2_O_2_ loss [30]. As shown in the dynamic analyses here, GOx therefore has limitations for stress testing when high concentrations are used and cell death is an outcome measure. However, GOx may remain useful for producing sustained lower H_2_O_2_ doses to probe sub-lethal effects of chronic oxidant exposure.

Another noteworthy observation with both pragmatic and biological implications was that ARPE-19 cells containing phagocytosed particles, including control beads, were more stress resistant than were cells that lacked particles. The pragmatic experimental caveat raised by this observation is that particle free cells are a poor control when analyzing the effect of phagocytosed melanosomes within cells. Beads were selected as the control particle for use here because both beads and melanosomes are indigestible and could have similar effects on the lysosomal compartment; as we have previously discussed, internalized particles such as those studied here are likely phagolysosomes [34]. The mechanism(s) whereby the process of phagocytosis or the presence of indigestible particles in phagolysosomes confers stress protection is unknown. Nor is it known whether different phagocytosed particles have different protective effects, although previous studies showed no particle-specific differences in several parameters relevant for stress protection, including lysosomal integrity, mitochondrial function, ATP levels, cell-substrate attachment, and expression levels of several antioxidant enzymes [20].

Aside from pragmatic issues related to the design of experiments involving phagocytosis to load cells with particles, there may be biological implications for RPE cells in situ arising from the observation that phagocytosis itself or the presence of indigestible particles within RPE cells can confer stress protection. A major function of the RPE within eyes is the daily phagocytosis of shed photoreceptor outer segments [39]. The content of largely indigestible granules varies among individual RPE cells within the monolayer [40] and changes with age as lipofuscin accumulates [41,42]. Ongoing phagocytosis and granule accumulation are usually considered detrimental effects of RPE aging, but perhaps that view is oversimplified.

Another provocative observation resulting from the dynamic cell death measures used here is that sites of early RPE cell death in a stressed culture monolayer appeared to serve as a nidus for the spread of death to adjacent cells. This outcome suggests that RPE cells do not die randomly when subjected to a universal stressor such as hydrogen peroxide. Coupling this observation with the observation that stress susceptibility is affected by discrete cell-cell differences (such as melanosome content), one might expect that oxidative stress over time would produce a pattern of cell death in the RPE monolayer that is focal or patchy, spreading from a peculiarly sensitive cell or cell patch. Interestingly, a non-uniform pattern of RPE cell death is a hallmark of one form of age-related macular degeneration: geographic atrophy (GA) [43].

In trying to understand why and how RPE oxidative stress contributes to retinal degenerative disease, research has focused on identifying widespread sources of stress to the RPE layer or on determining the content of antioxidants throughout the tissue. An alternative strategy might be to focus instead on individual RPE cells to determine what discrete, autonomous cell traits, such as their melanosome content, might modify their susceptibility to ubiquitous sources of stress like light or chemical oxidants. Whether the tissue is at risk may be determined not only by levels of stress exposure but also by the frequency or location of highly susceptible RPE cells.

As with all studies employing cell cultures, this investigation has limitations for extrapolating outcomes to the RPE tissue in situ. Phagocytosed melanosomes were used here to load cells with controlled numbers of pigment granules, but it is possible that phagocytosed organelles differ functionally from endogenous melanosomes since the former are likely encapsulated in phagolysosomal membranes. Perhaps the function of the phagocytosed granule is more closely related to that of endogenous melanolysosomes, which are complex granules with properties of both melanosomes and lysosomes that accumulate in the aging RPE [44]. Cell culture models, especially cell lines in early culture as used here, lack the polarized organizational state of RPE cells in situ. The largely apical distribution of endogenous melanosomes in the RPE of young eyes is therefore not recapitulated in culture models, where the granules are largely perinuclear. Properties of the melanosomes that are related to their sub-cellular position (such as optical light screening) may therefore be less efficient in vitro. Although positional effects cannot be ruled out, an antioxidant function to protect against H_2_O_2_–induced stress as studied here may be less affected by subcellular location of the granules.
